# Sacral Neuromodulation for Refractory Urge Incontinence Is Less Effective Following Spinal Surgery

**DOI:** 10.1100/tsw.2011.13

**Published:** 2011-01-18

**Authors:** Angela M. Arlen, Charles R. Powell, Karl J. Kreder

**Affiliations:** Department of Urology, University of Iowa, Iowa City, USA

**Keywords:** urinary incontinence, sacral neuromodulation, neurogenic bladder

## Abstract

Patients with neurogenic disorders and voiding dysfunction have been reported to respond poorly to sacral neuromodulation. We report on our experience in treating voiding symptoms with sacral neuromodulation after spinal surgery. The medical charts of patients evaluated for sacral neuromodulation from 2000–2008 were retrospectively reviewed. Indications, need for explantation, and clinical success (>50% symptom improvement) were recorded. The cohort of patients who had undergone prior spinal surgery was compared to patients with no history of spinal surgery or neurological disease. Thirty-two patients with a history of spinal surgery and 136 with no history of neurologic disease underwent sacral neuromodulation testing. Twenty men and women (62.5%) from the spinal surgery group ultimately underwent permanent implantation. Seventeen of the 32 patients were diagnosed with urge incontinence, of whom 52.9% reported a successful outcome at a mean of 2.3 years of follow-up, compared to an 80.3% success rate in patients with no history of spinal surgery (*p* = 0.018). Sixteen of 32 carried a diagnosis of urgency/frequency with 62.5% success at last follow-up, compared 73.9% (*p* = 0.35) of those without a history of spinal surgery or neurological disease. Thirteen of 32 patients diagnosed with urinary retention experienced a 61.5% long-term success rate, compared with 63.6% for those without spinal surgery and urinary retention. Six of 20 (30.0%) in the spinal surgery group were explanted at a mean time of 2.9 years, compared with 27 of 102 (26.5%) of the non-neurologic patients. Clinical success can be achieved using sacral neuromodulation in patients with voiding dysfunction and a history of spinal surgery; however, those with urge incontinence are less likely to report significant improvement.

## INTRODUCTION

Refractory irritative voiding symptoms, urge incontinence, and idiopathic urinary retention all pose a challenge to urologists. Neuromodulation has become a widely accepted treatment modality for various forms of voiding dysfunction, with success in treating both overactive bladder and urinary retention. The work of Tanagho and Schmidt in 1982 contributed to modern neurostimulation, and the indications for this therapy have continued to expand. Chronic pelvic pain, fecal incontinence, and interstitial cystitis have led to an increasingly diverse population undergoing sacral neuromodulation, many of whom have not been reported upon[1,2,3,4,5,6,7,8].

Voiding dysfunction is common among patients with neurological disease. The use of sacral neuromodulation for refractory voiding dysfunction has been reported in a variety of neurological disorders, with varied success[9,10,11,12,13]. Individuals with voiding dysfunction in the setting of spinal disease requiring surgery represent a unique patient population. Scheepens et al. reported in 2002 that individuals with a surgical history for intervertebral disk prolapse had an increased likelihood of positive response to percutaneous nerve evaluation[14]. However, to our knowledge, this is the first series to report on the success of sacral neuromodulation in patients with a history of spinal surgery.

## MATERIALS AND METHODS

Institutional Review Board approval was obtained for this retrospective review. Thirty-two patients with a history of spinal surgery and voiding dysfunction underwent sacral neuromodulation testing at our institution from May 2000 to February 2008. Outcomes of this cohort were compared to patients with no history of spinal or neurological disease who underwent sacral neuromodulation testing for similar indications. Two individuals in the spinal surgery cohort had diabetes, and other neurological diseases included one each with myofascial pain, restless leg syndrome, cerebrovascular accident, and migraine headaches. All patients underwent medical history, physical examination, and urodynamic studies in order to evaluate bladder function prior to neuromodulation testing. Medical charts were retrospectively reviewed for urodynamic findings, history of spinal surgery, indications for sacral neuromodulation, explantation, and clinical success of sacral neuromodulation on follow-up.

Those individuals undergoing sacral neuromodulation for urinary retention had evidence of acontractile bladder or detrusor hyposensitivity on urodynamic testing. Clinical success was defined as >50% symptom improvement from baseline per patient report; this measure was utilized both prior to permanent device implantation, and to monitor patient satisfaction and success at follow-up clinic appointments.

### Surgical Technique

All sacral neuromodulation devices were placed by a single surgeon (KJK) under either local or general anesthesia, depending on patient anatomy and preference. Prior to 2003, a percutaneous test lead was placed under fluoroscopic guidance. If successful during a test period, the percutaneous lead was removed and a four-contact permanent lead was placed at the same time as an implantable pulse generator. After 2003, the test stimulation procedure, or stage I, involved insertion of a needle and tined lead (insulated wire) into a sacral foramen (usually S3 or S4) on either side[4]. The second-stage procedure (approximately 1–2 weeks after implantation of the temporary device) was performed in all patients experiencing a >50% improvement in symptoms during the trial stimulation period.

## RESULTS

Thirty-two patients (16 females, 16 males) with a history of spinal surgery underwent sacral neuromodulation testing from May 2000 to February 2008. The average age was 56.03 years (range 30–77). Twenty patients (62.5%) went on to permanent implantation. Mean follow-up for all patients in both groups was 2.3 years. Indications for spinal surgery are listed in Table 1. The control group consisted of 136 patients with no history of spinal surgery or neurological disease; 102 (75%) of these patients went on to permanent device implantation.

**TABLE 1**
**Indications for Spinal Surgery**

| Indication for Spinal Surgery | No. of Patients |
|---|---|
| Disk disease | 22 |
| Tethered spinal cord | 2 |
| Malignancy of spinal cord | 1 |
| Polio | 1 |
| Neurofibroma of spinal cord | 1 |
| Lipoma of spinal cord | 1 |
| AVM of cervical spinal cord | 1 |
| Trauma | 1 |
| Spinal surgery of unknown type | 2 |

The successful conversion rate to permanent generator for patients with urge incontinence was 58.8%, significantly lower than the 89.5% of individuals with no history of neurological disease ($p$ = 0.002). There was a 75% conversion rate for patients suffering from urgency/frequency, comparable to the 71.4% rate in individuals without a neurological diagnosis. The conversion rate for urinary retention was 69.2% in those with previous spinal surgery and 68.2% with no neurological disorder (Table 2).

**TABLE 2**
**Successful Conversion Rate**

| | History of Spinal Surgery | Control | *p* Value |
|---|---|---|---|
| Urge incontinence | 58.8% | 89.5% | 0.002 |
| Urgency/frequency | 75.0% | 71.4% | 0.772 |
| Urinary retention | 69.2% | 68.2% | 0.948 |

During the follow-up period (mean 2.3 years), clinical success was defined as >50% symptom improvement per patient report. Those who failed the test stimulation were included for analysis as well. Of the patients with urge incontinence who had undergone previous spinal surgery, 52.9% reported a successful outcome compared to an 80.3% success rate in patients with no evidence of neurological disease or history of spinal surgery ($p$ = 0.018). Of patients with urgency/frequency, 62.5% reported >50% improvement following device implantation, compared to 73.9% ($p$ = 0.35) of those without spinal surgery or neurological disease. All patients with urinary retention were catheter-dependent preoperatively, performing clean intermittent catheterization a minimum of three times per day. Success in this group was defined as requiring catheterization once per day or less. Successful outcome with urinary retention was similar in both groups; 61.5% in those with a history of spinal surgery and 63.6% in those without (Table 3).

Six of 20 patients in the spinal surgery group ultimately had the permanent device removed, for an explantation rate of 30.0%. These devices were explanted at mean time to explantation of 2.9 years, which is comparable to the explantation rate of 27 of 102 (26.5%) in the control group with a mean follow-up of 2.95 years (Table 4). Two patients had the device removed for infection, two secondary to pain at the generator site, one due to lost efficacy, and one was removed secondary to dead battery. None of these differences achieved statistical significance.

**TABLE 3**
**Overall Success Rate**

| | History of Spinal Surgery | Control | *p* Value |
|---|---|---|---|
| Urge incontinence | 52.9% | 80.3% | 0.018 |
| Urgency/frequency | 62.5% | 73.9% | 0.35113 |
| Urinary retention | 61.5% | 63.6% | 0.90122536 |

**TABLE 4**
**Explantation Data**

| | Devices Explanted | *p* Value |
|---|---|---|
| History of spinal surgery | 6 (30%) | 0.74 |
| Control | 27 (26.5%) | |

## DISCUSSION

Although individuals with neurogenic disorders and voiding dysfunction have been reported to respond poorly to sacral neuromodulation, experience with patients suffering from voiding dysfunction following spinal surgery has not been described[9,10,11,12]. Hohenfellner and colleagues report 44% success during the test phase treating patients with a neurologic etiology for voiding dysfunction[11]. Nine of these patients suffered partial or complete spinal cord injuries, but none were reported to have undergone spinal surgery. Only one patient in our series received surgery for a spinal cord injury and she failed to improve after test stimulation. Lombardi and Del Popolo provided a larger experience of spinal cord injured patients, followed over 61 months, and this work suggests that excellent results can be achieved using sacral neuromodulation to treat partial spinal cord injuries[12].

Patients with refractory voiding dysfunction in the setting of spinal disease requiring surgery represent a unique patient population. Voiding dysfunction can be multifactorial, with narcotic use and decreased ambulation playing a role. In order to mitigate the impact of these factors, all patients waited a minimum of 6 months after spinal surgery to undergo urodynamic testing. Spinal cord injury is a leading cause of neurogenic bladder dysfunction, and urgency/urge incontinence and urinary retention are frequently documented in individuals with a history of spinal surgery. The presence of spinal surgery in the current investigation decreased the likelihood of a successful outcome after sacral neuromodulation for urge incontinence, whereas patients with urgency/frequency and urinary retention had similar success rates as those individuals without surgery on the bony spine. Amundsen and colleagues described 52% conversion from the test phase and the presence of a neurologic condition predicted a lower cure rate[13]. It is interesting to note that in this investigation, 41% in the younger age group and 21% in the older group had undergone spinal surgery of some kind. It appears that surgery on the bony spine may not only affect voiding function, but also the likelihood of success of a proven treatment modality for refractory urge incontinence.

Limitations include the retrospective study design and the potential for recall bias. In addition, the average length of follow-up for all patients was only 2.3 years. Patients are scheduled for initial postoperative check at 4–6 weeks, and are scheduled for annual or biannual appointments thereafter. Those who are satisfied with their symptom control often return only for battery exchanges rather than on a scheduled basis. Finally, it is difficult to ascertain whether patients had voiding dysfunction in the presence of or secondary to spinal surgery, as baseline urodynamics studies were not obtained prior surgery on the spine.

## CONCLUSIONS

Voiding dysfunction following spinal surgery can be treated successfully with sacral neuromodulation, but success is less likely in patients suffering from urge incontinence. Potential sacral neuromodulation candidates need to be counseled accordingly.
